# ﻿*Sinocrassula cuonaensis* (Crassulaceae), a new succulent species from Southern Xizang, China

**DOI:** 10.3897/phytokeys.263.159143

**Published:** 2025-09-24

**Authors:** Xiang-Dong Qiu, Jia-Jing Shi, Ya-Lin Feng, Feng-Di Zhang, Sheng-Wei Wang, Neng Wei, Qing-Feng Wang

**Affiliations:** 1 School of Ecology and Environment, Tibet University, Lhasa, Xizang 850032, China Tibet University Lhasa China; 2 State Key Laboratory of Plant Diversity and Specialty Crops, Wuhan Botanical Garden, Chinese Academy of Sciences, Wuhan, Hubei 430074, China Wuhan Botanical Garden, Chinese Academy of Sciences Wuhan China; 3 University of Chinese Academy of Sciences, Beijing 100049, China University of Chinese Academy of Sciences Beijing China; 4 Sino-Africa Joint Research Center, Chinese Academy of Sciences, Wuhan, Hubei 430074, China Sino-Africa Joint Research Center, Chinese Academy of Sciences Wuhan China

**Keywords:** Biodiversity hotspot, phylogeny, Qinghai-Xizang Plateau, taxonomy

## Abstract

*Sinocrassula cuonaensis*, a new species of Crassulaceae from southern Xizang, is described and illustrated based on molecular and morphological evidence. Phylogenetically, it is sister to *S. densirosulata* with robust support. Morphologically, this new species is similar to S. indica var. viridiflava, *S. densirosulata*, and *S. jiaozishanensis* in having glabrous indumentum and long inflorescences, but it can be easily distinguished by its rosulate, thick, and near-cylindrical basal leaves, greenish white flowers, and sub-rectangular nectar scales. A key for identification of the genus, including the new species, is provided.

## ﻿Introduction

Crassulaceae by Saint-Hilaire (1805: 123) is the largest family in Saxifragales, comprising 34 genera and ca. 1400 species, and is divided into three subfamilies (Crassuloideae, Kalanchoideae, and Sempervivoideae) (Thiede and Eggli 2007; Messerschmid et al. 2020). Among the genera in Crassulaceae, *Sinocrassula* A.Berger in Berger (1930: 462) is a small genus with 14 species that belongs to Sempervivoideae. The main diagnostic features of *Sinocrassula* are pentamerous flowers, a single whorl of stamens, basally connate carpels, and basal leaves often arranged in a rosette. The genus is sister to *Kungia* K.T.Fu in Fu (1988: 3); together, these two genera form a clade sister to the clade containing *Orostachys* Fisch. in Fischer (1809: 270), *Hylotelephium* H.Ohba in Ohba (1977: 46), and *Meterostachys* Nakai in Hara (1935: 74) in Telephieae (Messerschmid et al. 2020; Liu et al. 2023). Most species of *Sinocrassula* are distributed in East Asia (China) and South Asia (India, Pakistan, Nepal, Bhutan, and Sikkim), with only *S. vietnamensis* Aver. & V.V. Byalt. (2014: 348) distributed in Southeast Asia (North Vietnam) (Fu and Ohba 2001; Wang et al. 2013; Averyanov et al. 2014; Wang et al. 2022; Li et al. 2024; Xu et al. 2025; Li et al. 2025). Beyond their geographical distribution, some species of *Sinocrassula* have horticultural and medicinal uses. Horticulturally, *S. yunnanensis* (Franch.) A. Berger in Berger (1930: 463) is cultivated as a succulent plant and is fairly popular in markets, whereas pharmacologically, *S. indica* (Decne.) A. Berger in Berger (1930: 463) has long been used in traditional Chinese medicine (Xie and Yoshikawa 2012; Zhang et al. 2014).

In field trips conducted in August 2023 and in October 2024, we collected specimens of *Sinocrassula* from two populations in Cuona City, Xizang Autonomous Region, China. Field-based morphological analysis indicated that the characters of basal leaves and flowers in these specimens significantly differed from those of other members of *Sinocrassula*. Through comparative morphological studies and molecular phylogenetic analyses, we further confirmed and described these specimens here as a new species, *S. cuonaensis*.

## ﻿Materials and methods

Specimens of the new species were collected from southern Xizang, with their living materials cultivated at the greenhouse of Wuhan Botanical Garden, Chinese Academy of Sciences. Morphological characteristics were observed and photographed with a Zoom Stereo Microscope-TS-10W stereomicroscope (PDV, China) and an EOS M5 Canon camera and were measured using ImageJ (https://imagej.nih.gov/ij/). Morphology of seeds was observed and photographed with a HITACHI TM3030 scanning electron microscope (SEM) (Hitachi, Ltd., Japan). One sample of the new species and 39 other Crassulaceae samples were used to reconstruct phylogenetic trees to determine the phylogenetic position of the new species. Among these 40 samples, 37 were from Sempervivoideae (including 15 accessions in *Sinocrassula*), and 3 were from Kalanchoideae. According to previous studies (Messerschmid et al. 2020; Xu et al. 2025), the samples from Kalanchoideae were employed as the outgroup. The sources of the materials and their corresponding GenBank accession numbers are listed in Table 1.

**Table 1.** List of taxa sampled with information related to taxonomy, GenBank accession numbers, and references.

Species	Location	Voucher specimen	*matK*	*rbcL*	*psbA*-*trnH*	*trnL*-*trnF*	ITS	Reference
*Sinocrassula cuonaensis*	Xizang, China	*Wei & Xin WN43* (HIB, PE)	PV610727	PV610839	PV641593	PV641595	PV548041	This study
*S. ambigua*	Yunnan, China	*Chen et al. YUS12973* (YUKU)	PQ629047	PQ629039	PQ629054	PQ629032	PQ611189	Xu et al. 2025
*S. ambigua*	Yunnan, China	*Chen et al. YUS6698* (YUKU)	PQ629048	PQ629040	PQ629059	PQ629035	PQ611190	Xu et al. 2025
*S. ambigua*	Yunnan, China	*Chen et al. YUS12672* (YUKU)	PQ629046	PQ629038	PQ629055	PQ629030	PQ611188	Xu et al. 2025
*S. densirosulata*	Sichuan, China	*Chang XC19075* (SC)	MW206800	MW206800	MW206800	MW206800	–	Chang et al.2021
*S. holotricha1*	Sichuan, China	*Zhao et al. YUS13475* (YUKU)	PQ629050	PQ629042	PQ629056	PQ629034	PQ611192	Xu et al. 2025
*S. holotricha2*	Sichuan, China	*Zhao et al. YUS12867* (YUKU)	PQ629051	PQ629043	PQ629057	PQ629031	PQ611193	Xu et al. 2025
*S. indica*	Yunnan, China	zjq20160061	MN794334	MN794334	MN794334	MN794334	–	Zhao et al. 2020
S. indica var. viridiflava	Hunan, China	*Jiang et al. 0911* (HIB)	PV610728	PV610840	PV641594	PV641596	–	This study
*S. jiaozishanensis*	Yunnan, China	*Chen et al. JZS001* (YUKU)	MZ343261	MZ343263	MZ343262	MZ343264	MZ343260	Wang et al. 2022
*S. jiaozishanensis*	Yunnan, China	*Chen et al. JZS002* (YUKU)	MZ343266	MZ343268	MZ343267	MZ343269	MZ343265	Wang et al. 2022
*S. yunnanensis*	Yunnan, China	*Chen et al. YUS13776* (YUKU)	PQ629049	PQ629041	PQ629061	PQ629033	PQ611191	Xu et al. 2025
*S. yunnanensis*	Yunnan, China	*Chen s.n.* (HIB)	KC988295	–	–	–	KC988288	Chen et al. 2014
*S. yunnanensis*	Yunnan, China	*Mayuzumi C00115* (TI)	–	–	–	AB480669	AB088582	Mayuzumi and Ohba 2004
*S. yunnanensis*	Yunnan, China	*Chen et al. YUS6697* (YUKU)	PQ629053	PQ629045	PQ629060	PQ629037	PQ611195	This study
*Kungia aliciae*	China	*Mayuzumi CH00061* (TI)	–	–	–	AB480632	AB480591	Mayuzumi and Ohba 2009
*Adromischus fallax*	Saudi Arabia	*Bruyns 2997* (BOL)	MH50364	–	LN878728	LN878814	MH503497	Bruyns et al. 2019
*Aeonium decorum*	Gomera, Spain	*Mort 1435* (WS)	AY082165	–	AY082197	AY082239	AY082130	Mort et al. 2002
*Aichryson pachycaulon*	Spain	*Mort 1404* (WS)	AY082157	–	AY082182	AY082223	AY082105	Mort et al. 2002
*Cotyledon barbeyi*	Kenya	*Bruyns 12754* (BOL)	MH503487	–	–	MH503217	MH503623	Bruyns et al. 2019
*Dudleya pulverulenta*	Mexico	*Oceguera s.n.* (XAL)	–	–	–	–	EF632171	Messerschmid et al. 2020
*Echeveria amoena*	Mexico	*Carrillo-Reyes & Nicolalde 4233* (IEB, XAL)	–	–	–	–	EF632172	Messerschmid et al. 2020
*Graptopetalum amethystinum*	Mexico	*Acevedo 1734* (NYBG, XAL)	–	–	–	–	AY545690	Messerschmid et al. 2020
*Hylotelephium tatarinowii*	China	*Zhang 100717 – 08* (PEY)	–	–	KF113734	KF113787	KF113681	Zhang et al. 2014
*Kalanchoe gracilipes*	Madagascar	*Bruyns 6232* (BOL and MO)	MH503489	–	–	MH503219	MH503625	Bruyns et al. 2019
*Lenophyllum acutifolium*	New York Botanical Garden	*Rose s.n.* (NYBG)	–	–	–	–	AY545709	Messerschmid et al 2020
*Meterostachys sikokianus*	Nagasaki, Japan	*Mayuzumi et al. C0028* (TI)	–	–	–	AB480670	–	Mayuzumi and Ohba, unpublished
*Monanthes adenosepes*	Tenerife, Spain	*Santos s.n.*	AY082264	–	AY082277	AY082291	AY082118	Mort et al. 2002
*Orostachys malacophylla*	Primorsky, Russia	*Mayuzumi CH00054B* (TI)	–	–	–	AB480617	AB480580	Mayuzumi and Ohba, unpublished
*Pachyphytum fittkaui*	Mexico	*HBG-49458*	–	–	–	–	FJ753925	Messerschmid et al. 2020
Petrosedum amplexicaule subsp. tenuifolium	Spain	*M.J.G 024790*	MT181567	–	–	MT336100	–	Messerschmid et al. 2020
*Phedimus aizoon*	Hebei, China	*Zhang et al. 120613-03* (PEY)	–	–	KF113735	KF113788	KF113682	Zhang et al. 2014
*Prometheum chrysanthum*	Turkey	*Stephenson 4R022*	KX45252.1	–	–	–	HE999634	Messerschmid et al. 2020
*Pseudosedum lievenii*	Turkmenistan	*Regel 1079* (US)	–	–	–	–	KJ569920	Zhang et al. 2014
*Rhodiola humilis*	Xizang, China	*Zhang et al. 110804-03-03* (PEY)	KP114838	KP115042	KP114937	KP115148	KP114742	Zhang et al. 2014
*Sedum alfredii*	China	*Kokubugata 17191* (TNS)	–	–	–	LC229500	AB930260	Messerschmid et al. 2020
*Sempervivum tectorum*	Canada	*CCDB-18313-F08*	–	MG249291	–	–	MG237296	Kuzmina et al. 2017
*Thompsonella colliculosa*	Mexico	*Carrillo-Reyes & Perez-Calix 2714* (GUADA, IBUG, and IEB)	–	–	–	–	EF632177	Messerschmid et al. 2020
*Umbilicus schmidtii*	Cape Verde	*Romerias & Carine 3170* (LISC)	KP279381	–	KP279450	KP279339	–	Romerias et al. 2015
*Villadia diffusa*	Mexico	*Nicolalde 1461* (XAL)	–	–	–	–	FJ753973	Messerschmid et al. 2020

We selected four plastid markers [(*matK*, *rbcL*, *psbA*-*trnH*, and *trnL*-*trnF*) and one nuclear marker (internal transcribed spacer, ITS) for amplification and sequencing. The primer design and PCR condition settings followed Kress and Erickson (2007) and Zhang et al. (2015). Alignments were visually and manually checked in Geneious v8.0.2 (Kearse et al. 2012). To ensure compatibility of the ITS marker and four plastid marker datasets, an incongruence length difference (ILD) test (Farris et al. 1995) was conducted, which is implemented in PAUP v4.0a169 (Swofford 1998). The ILD test was run with 1000 replications, each using a heuristic search with 100 random addition sequence replicates and TBR branch swapping; refer to Yu et al. (2022). The ILD test was performed using a significance level of p = 0.01, following Cunningham (1997). The phylogenetic trees were reconstructed using maximum likelihood (ML) analysis in IQ-TREE v1.6.8 (Nguyen et al. 2015) and Bayesian inference (BI) analysis in MrBayes v3.2.6 (Ronquist et al. 2012). PartitionFinder 2 (Lanfear et al. 2017) was applied to choose the best-ﬁt model under the Bayesian information criterion (BIC). The customization of the phylogenetic tree was completed on the online program iTOL (Letunic and Bork 2007). For detailed methods in phylogenetic tree reconstruction, refer to Wei et al. (2021).

## ﻿Results and discussion

### ﻿Morphological study

The new species *Sinocrassula cuonaensis* possesses fleshy basal rosette leaves, a glabrous surface, long inflorescences, and greenish white flowers, which are distinct from other species in *Sinocrassula*. It is morphologically most similar to S. indica var. viridiflava K.T.Fu in Fu (1974: 606), *Sinocrassula densirosulata* (Praeger) A.Berger (1930: 463), and *S. jiaozishanensis* Chao Chen, J.Guan Wang & Z.R.He in Wang et al. (2022: 13), differing mainly in its leaf blade, inflorescence length, flower color, and nectar scales. In leaf morphology, the basal leaves of *S. cuonaensis* are densely rosulate and nearly cylindrical, contrasting with the spatulate-oblong leaves of S. indica var. viridiflava, the spatulate leaves of *S. densirosulata*, and the opposite leaves of *S. jiaozishanensis*, which lack a rosette arrangement. With respect to inflorescence length, *S. cuonaensis*, S. indica var. viridiflava, and *S. jiaozishanensis* exhibit inflorescences exceeding 15 cm, while *S. densirosulata* deviates from this pattern. Regarding flower color, *S. cuonaensis* displays greenish-white flowers, distinct from the greenish-yellow flowers of S. indica var. viridiflava, the purplish red flowers of *S. densirosulata*, and the reddish-purple flowers of *S. jiaozishanensis*. In terms of nectar scale morphology, *S. cuonaensis* exhibits wider nectar scales (ca. 0.7 mm), while S. indica var. viridiflava (ca. 0.5 mm) and *S. jiaozishanensis* (ca. 0.3 mm) have narrower scales. The distinguishing characteristics of these four species are listed in detail in Table 1.

### ﻿Molecular phylogenetic analyses

**Figure 1.**
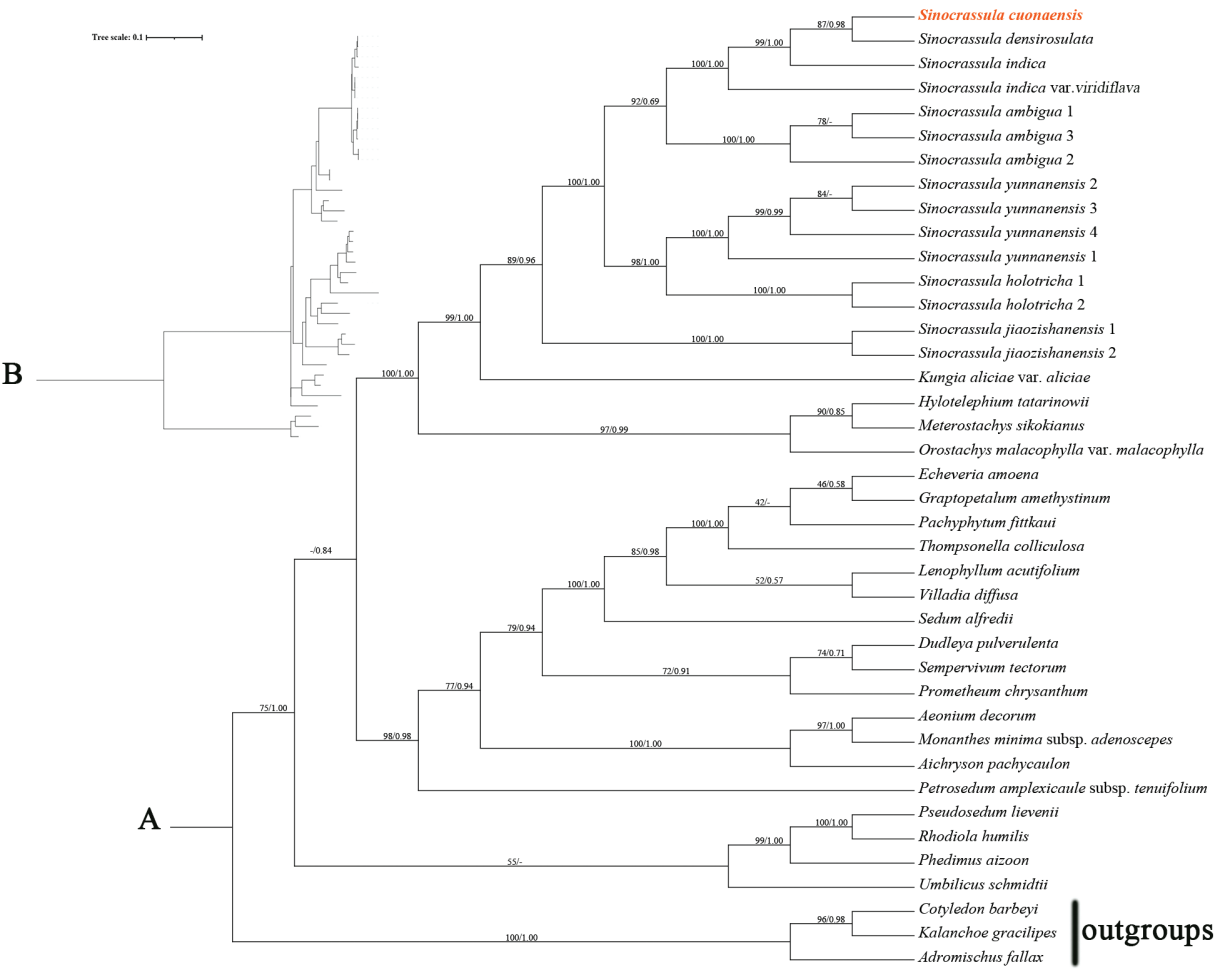
A. The maximum likelihood phylogenetic tree of *Sinocrassula cuonaensis* and its allies based on the combined dataset of ITS, *rbcL*, *matK*, *psbA*-*trnH*, and *trnL*-*trnF*. The new species is highlighted in bold and red; B. The same tree as A. Showing branch lengths proportional to nucleotide substitutions per site.

The ILD test showed that there is no signiﬁcant incongruence (p = 0.997, >0.01) between the ITS and four plastid markers (*matK*, *rbcL*, *psbA*-*trnH*, and *trnL*-*trnF*), supporting their combination in the following phylogenetic inference. The models GTR+I+G, TVM, K81UF+I, K81UF+G, and GTR+G were each selected as the best-fit models for the ITS, *matK*, *rbcL*, *psbA*-*trnH*, and *trnL*-*trnF* datasets, respectively, to infer the ML tree and Bl tree. As shown in Fig. 1, the ML and BI trees are identical, and both bootstrap support (BS) values and posterior probabilities (PP) for each clade are listed on the ML tree. The 37 accessions from Sempervivoideae are revealed as monophyletic with moderate support (BS = 75%, PP = 1). Species from *Sinocrassula* together are also revealed as monophyletic, though not fully supported (BS = 89%, PP = 0.96), with *Kungia* as the sister genus with strong support (BS = 99%, PP = 1). The new species *Sinocrassula cuonaensis* and *S. densirosulata* formed a clade with strong support (BS = 87%, PP = 0.98), supporting their sister relationship. The clade containing these two species is sister to *S. indica*, then to S. indica var. viridiflava, forming a strongly supported clade (BS = 100%, PP = 1).

### ﻿Discussion

Integrating morphological and phylogenetic analyses confirmed *Sinocrassula cuonaensis* as a new species, with molecular evidence identifying *S. densirosulata* as its sister taxon. Morphologically, the new species is easily distinguishable from *S. densirosulata*. Although both possess rosette leaves, their leaf shape, inflorescence length, and flower color are different from each other. The new species is also closely related to *S. indica* and S. indica var. viridiflava. Both the new species and *S. indica*, as well as S. indica var. viridiflava, exhibit rosette leaves and long inflorescences, but they can be distinguished by leaf color, flower color, and nectar scale morphology. In our phylogeny, two accessions of *S. jiaozishanensis* formed the first divergent clade in *Sinocrassula*. This result is consistent with Wang et al. (2022) and Xu et al. (2025). This discovery of the new species further highlights the ignored species diversity of *Sinocrassula* in China, with a number of new species discovered and named in recent years (Wang et al. 2022; Li et al. 2024, 2025; Xu et al. 2025). The new species has been only documented in southern Xizang (Tibet), which is located in the Himalayan biodiversity hotspot. The discovery of this new species not only enriches the floristic records in the region but also provides valuable materials for studying the biogeography of *Sinocrassula*.

### ﻿Taxonomic treatment

#### ﻿ Sinocrassula cuonaensis

Taxon classificationPlantaeSaxifragalesCrassulaceae

N.Wei & Q.F.Wang sp. nov.

8EA73754-9A59-595A-AD1F-4B4FB1F895E6

urn:lsid:ipni.org:names:77369656-1

Figs 2, 3

##### Type.

China • Xizang: Cuona [Tsona] City, Lebu Valley, elev. 2826 m, 27.864222, 91.796976, on the moist rocks on valley slopes by the roadside, 3 Aug. 2023, *N.Wei & Z.H.Xin WN43* (holotype: HIB!; isotypes: HIB!, PE!).

**Figure 2.**
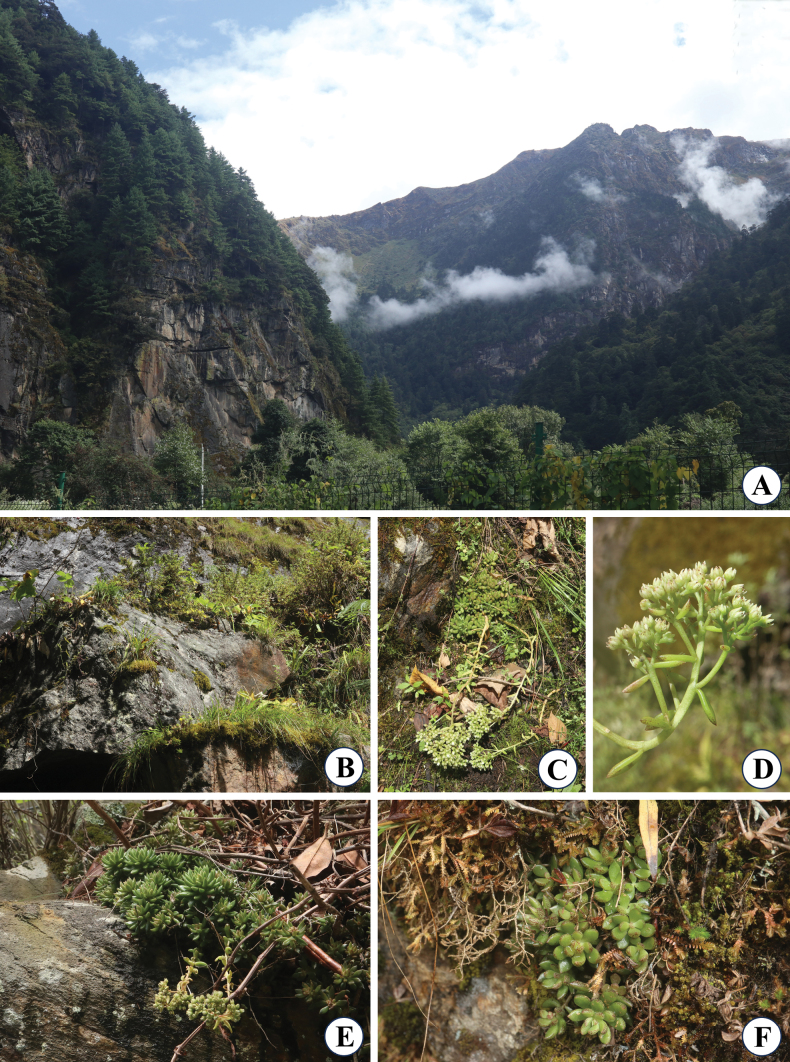
*Sinocrassula cuonaensis*. A. Its habitat in a deep valley; B. Its rocky habitat; C. The whole individuals; D. A flowering branch; E. The whole individuals of mature plants; F. The whole individuals of seedlings. [Voucher specimens: *X.D.Qiu et al. XZ08* (HIB!)].

##### Diagnostic description.

*Sinocrassula cuonaensis* is similar to S. indica var. viridiflava, *S. densirosulata*, *S. jiaozishanensis* in having glabrous plant and long inflorescence, but its rosulate, thick, and near-cylindrical basal leaves (vs. rosulate, spatulate, not rosulate, respectively) and greenish white flowers (vs. greenish yellow, purplish red, reddish purple, respectively) differ from the others. Besides, this new species has sub-rectangular nectar scales that are similar to S. indica var. viridiflava (vs. near-rectangular oblong) and *S. densirosulata* (vs. spatulate-quadrate) but different from *S. jiaozishanensis* (vs. oblong) (Table 2).

**Table 2.** Comparison of *S. cuonaensis* and its morphologically similar species (- indicates the missing information).

Character	*S. cuonaensis*	S. indica var. viridiflava	*S. densirosulata*	*S. jiaozishanensis*
Life cycle	Biennial	Annual	Annual	Perennial
Basal leaves	Rosulate, thick, and near-cylindrical	Rosulate, spatulate-oblong	Rosulate, spatulate	Not rosulate, opposite
Plant surface	Glabrous	Glabrous	Glabrous	Glabrous
Stem leaves	Obovate	Oblanceolate	Spatulate to elliptice	Round obovate or oblanceolate to oblong
Stem leaves base	Gradually narrowing	Gradually narrowing	Rounded to cuneate	-
Stem leaves apex	Abruptly acute	Acute	Acuminate	Acute or cuspidate
Bracts	Elliptic or anceolate	Linear-lanceolate	–	Obovate or lanceolate
Inflorescence	15–26 cm long	13–20 cm long	1.5–3 cm long	15–20 cm long
Flowers color	Greenish white	Greenish yellow	Purplish red	Reddish purple
Nectar scales	Sub-rectangular	Near-rectangular oblong	Spatulate-quadrate	Oblong
Nectar scales size	Ca. 0.7 × 0.5 mm	Ca. 0.5 × 0.7 mm	–	Ca. 0.3 × 0.6 mm
Phenology	August-November	September-October	July–November	March-June

**Figure 3.**
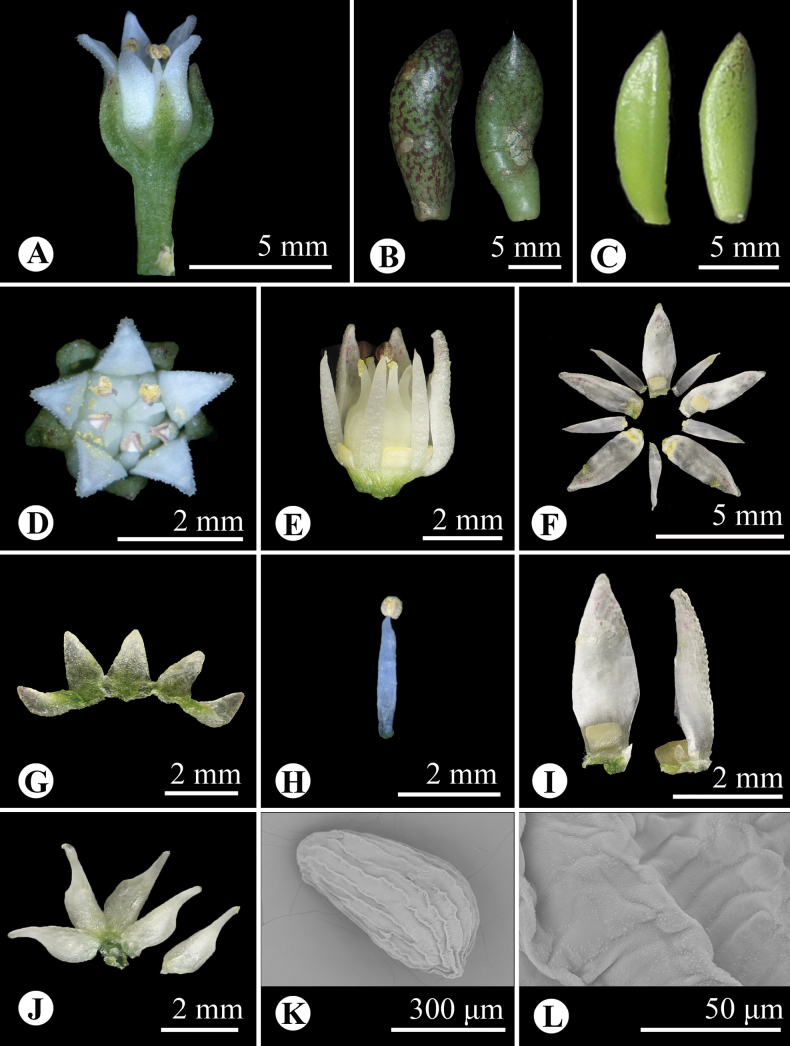
*Sinocrassula cuonaensis*. A. Flowers; B. Basal leaves; C. Stem leaves; D. Flower (top view); E. Flower with sepals and one petal removed (side view); F. Flower structure (with sepals and carpels removed); G. Sepals; H. Stamen; I. Petals and nectar scales; J. Carpels; K, L. Seeds under the scanning electron microscope. [*N.Wei & Z.H.Xin WN43* (HIB)]. According to the description, flowering and fruiting are at different times of the year. If so, these pictures belong to two different gatherings.

##### Description.

Biennial herbs, lithophytic or terrestrial, 10–30 cm tall, glabrous throughout. Roots fibrous. Leaves with basal rosette, oblanceolate to obovate, 0.9–2.2 × 0.4–0.8 cm, thickness 3–4 mm wide, apex mucronate apiculate to obtuse, green or surface reddish-brown, fleshy. Stem leaves alternate, oblanceolate, 1.2–1.7 × 0.3–0.4 cm, thickness 1.5–2.5 mm wide, apex mucronate apiculate, green, fleshy. Flowering stems 15–26 cm long, creeping or somewhat pendulous. Inflorescence corymbiform panicle, ca. 1.5–5.3 cm in diameter; bracts ca. 1 × 0.4 cm, green or sometimes with red. Flowers urceolate, ca. 7.5–8.0 × 3.4–3.6 mm, greenish white, fleshy. Sepals 5, ovate-triangular, apex obtuse, ca. 1.5 × 1.1 mm, green. Petals 5, ovate-lanceolate, minutely papillate abaxially, apex usually reflexed, ca. 3–4 × 1 mm, white. Stamens 5, ca. 3 mm long; anthers orbicular, ca. 2.5 mm long, pollen yellow. Nectar scales 5, rectangular, ca. 0.7 × 0.5 mm. Carpels 5, apocarpous, ovate-triangular, ca. 3 mm. Styles erect, ca. 1 mm long. Seeds ovoid-cylindric, ca. 0.50 × 0.25 mm, surface with longitudinal ribs and transverse ridges.

##### Phenology.

Flowering from August to September.

##### Distribution and habitat.

**Figure 4.**
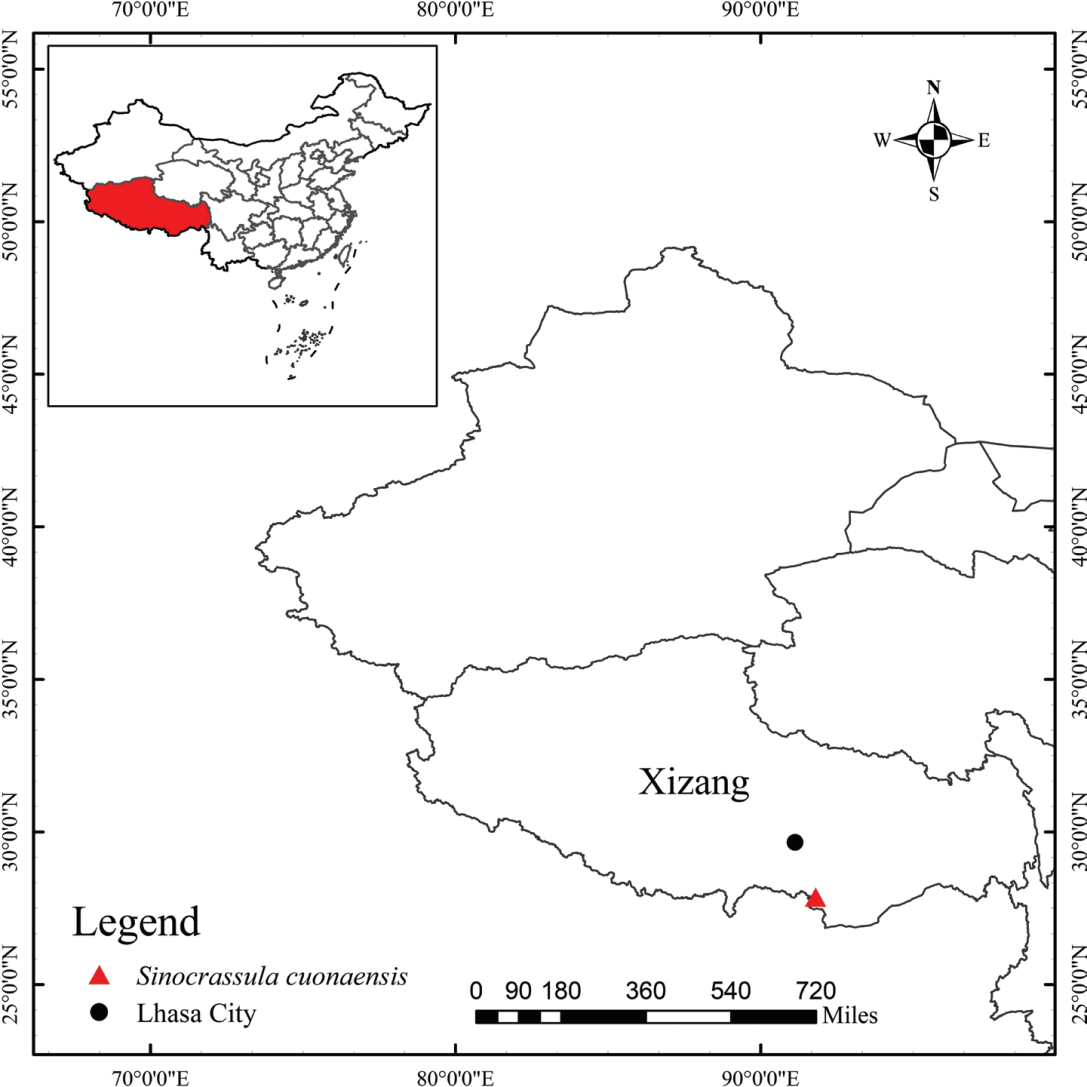
Geographical distribution of *Sinocrassula cuonaensis* in Cuona City, Xizang, China.

*Sinocrassula cuonaensis* is currently known only from southern Xizang, China (as depicted in Fig. 4). This new species was found on moist rocks along slopes in the valley at elevations of 2500–2850 m. The general vegetation condition of the habitat is characterized by the tree layer dominated by *Pinus wallichiana* A.B.Jacks., *Picea spinulosa* (Griff.) A.Henry, and *Cotoneaster* sp.; the shrub layer is dominated by shrubs of the families Urticaceae and Asteraceae; and the herb layer is primarily composed of *Pteridium latiusculum* (Desv.) Hieron., *Corallodiscus* sp., and *Impatiens scabrida* DC. Additionally, due to the steep topography, these rock walls are less disturbed by human activities.

##### Additional specimens examined

**(paratypes).** China • Xizang Autonomous Region: Cuona [Tsona] City, Lebu Valley, 27.85674995°N, 91.79360947°E, alt. 2714 m, 2 October 2024, *X.D.Qiu et al. XZ08* (HIB!); • in the same area, 27.83405975°N, 91.76442437°E, alt. 2502 m, 2 October 2024, *X.D.Qiu et al. XZ12* (HIB!).

##### Conservation status.

We found two populations with over 50 individuals in the type locality of *Sinocrassula cuonaensis*. The similar habitats have been thoroughly surveyed in the vast southern region of Xizang and the surrounding Bhutan and India. Further surveys in these regions could potentially reveal additional populations and individuals yet to be recorded. Therefore, we temporarily classify the species as Data Deficient (DD) according to the IUCN Red List Categories and Criteria (IUCN 2024).

##### Etymology.

The specific epithet ‘*cuonaensis*’ refers to Cuona City, where the species was discovered.

A key for identification of the genus, including the new species, is compiled and provided below.

### ﻿Key to the species of *Sinocrassula* A. Berger

**Table:** 

1	Plants usually more than 10 cm	**2**
–	Plants usually less than 10 cm	**12**
2	Leaves not with basal rosette	**3**
–	Leaves with basal rosette	**4**
3	Plant hairy	**1. *S. holotricha***
–	Plant glabrous	**2. *S. jiaozishanensis***
4	Lateral branches of inflorescence to 10 cm long	**3. *S. longistyla***
–	Lateral branches of inflorescence not to 10 cm long, usually 5–6 cm or shorter	**5**
5	Leaves slender linear lanceolate, apex acuminate	**4. *S. papillosa***
–	Leaves not slender linear lanceolate	**6**
6	Inflorescences usually 1.5–3.5 cm in diameter	**7**
–	Inflorescences usually more than 3.5 cm in diameter	**9**
7	Leaves asymmetrical	**5. *S. obliquifolia***
–	Leaves not asymmetrical	**8**
8	Basal leaves ovate to ovate-lanceolate, 0.7–1.1 × 0.3–0.5 cm	**6. *S. stenosquamata***
–	Basal leaves orbicular-lanceolate, 1–3 × 0.9–1.0 cm	**7. *S. ganluoensis***
9	Stem leaves dimorphic: proximal ones broadly obovate or elliptic, 2.0–2.7 cm wide, distal ones narrowly lanceolate to linear-lanceolate, 0.3–0.8 cm wide	**8. *S. diversifolia***
–	Stem leaves not dimorphic	**10**
10	Basal leaves oblanceolate to obovate, less than 0.8 cm wide; 3–4 mm thick; flowers greenish white	**9. *S. cuonaensis***
–	Basal leaves usually more than 0.8 cm wide; 2–3 mm thick; flowers white, red, yellow, or greenish yellow	**11**
11	Flowers red, yellow, or greenish yellow; sepals broadly triangular, ca. 1 mm wide; carpel 2.5–3.0 mm long, style less than 1 mm long	**10. *S. indica***
–	Flowers white; sepals narrowly triangular, 1.2–1.5 mm wide; carpel about 3.5 mm long, style 1.0–1.2 mm length	**11. *S. vietnamensis***
12	Plants densely shortly white pubescent	**12. *S. yunnanensis***
–	Plants glabrous, or sparsely hairy on leaves adaxially or on flowering stems	**13**
13	Leaves hairy, but flowering stems glabrous	**10. *S. indica***
–	Leaves glabrous, but flowering stems sometimes sparsely pilose	**14**
14	Sepals equaling or longer than petals; caudex branched, 3–6 cm long	**13. *S. ambigua***
–	Sepals shorter than petals; caudex not elongated	**15**
15	Basal leaves in a compact rosette; petals triangular-lanceolate	**14. *S. densirosulata***
–	Basal leaves caducous; petals oblong	**15. *S. techinensis***

## Supplementary Material

XML Treatment for Sinocrassula cuonaensis

## ﻿Additional information

### Use of AI

No use of AI was reported.

### Author ORCIDs

Xiang-Dong Qiu https://orcid.org/0009-0007-2464-6921

Jia-Jing Shi https://orcid.org/0009-0000-5808-2908

Sheng-Wei Wang https://orcid.org/0000-0002-8785-5065

Neng Wei https://orcid.org/0000-0002-0313-3912

Qing-Feng Wang https://orcid.org/0000-0001-9143-8849

### Data availability

All of the data that support the findings of this study are available in the main text.
